# Relationship between patients and medical professionals: expectations towards healthcare services in Nigeria

**DOI:** 10.1097/MS9.0000000000001505

**Published:** 2023-11-16

**Authors:** Abdullahi Tunde Aborode, Soyemi Toluwalashe, Christian Inya Oko, Tayo Nafisat Folorunso, Samuel Chinonso Ubechu, Rawa Badri, Edima Ottoho, Gabriel Nku Odok, Ayyatullahi Bolanle Kamaldeen, Abdulhammed Opeyemi Babatunde, Esther Oluwadamilola Olorunshola, Babatunde Shuaib Anidu, Seto Charles Ogunleye, Mercy Mayowa Olorunshola

**Affiliations:** a Healthy Africans Platform, Research and Development; b Clinical Laboratory Physician and Senior Resident, University College Hospital; c Department of Medicine and Surgery Faculty of Clinical Sciences, College of Medicine University of Ibadan, Ibadan; d Department of Medicine, Lagos state University College of Medicine, Lagos; e Al Hikmah University, Ilorin, Kwara State, Nigeria; f Department of Health Research, University of Lancaster, Lancaster, Lancashire, UK; g Department of Kinesiology, University of Louisiana at Lafayette, Lafayette, LA; h School of Public Health, Yale University, New Haven, CT; i Mycetoma Research Centre; j Faculty of Medicine, University of Khartoum, Khartoum, Sudan; k Boston University School of Public Health, Boston, MA; l Kamfo Anokye Teaching Hospital, Kwame Nkrumah University of Science and Technology, Kumasi, Ghana; m Department of Integrative Biology and Physiology, University of Minnesota Medical School, Minneapolis, MN; n Comparative Biomedical Sciences, College of Veterinary Medicine, Mississippi State University, Mississippi State, MS; o State University of New York at Binghamton, Binghamton, NY

*Dear Editor*,

Understanding patients’ response to healthcare and their expectations from medical professionals is a necessary bedrock for the success to healthcare delivery in Nigeria. However, the perception of healthcare providers in Nigeria, as well as the reception of healthcare services by patients, can exhibit variability due to a multitude of factors. These range from personal experiences and cultural conventions to socioeconomic status and the overall condition of the healthcare infrastructure. A breach in this healthcare delivery can affect how patients perceive healthcare and ultimately impact how they respond. When patients’ expectations are met, they are more likely to have a positive response ranging from increased satisfaction, adherence to medications, and trust in their doctors. Conversely, if this expectations are not met, there is less trust and confidence, reduced adherence and even discontinuity in treatments.

The duration of waiting time in outpatient clinics in Nigeria has been seen to be lengthy, leading to a prevailing sentiment of discontentment over the quality of medical treatments rendered at these healthcare facilities^1^. The absence of a time-specific appointment system has been proposed as a potential explanation for this phenomenon. Generally, outpatient services in Nigeria do not involve scheduling appointments at designated times, thereby encouraging a significant number of patients to attend clinics without prior appointments, particularly during the morning hours, leading to a situation where physicians are faced with excessive workload and patients have extended waiting periods before they can interact with their healthcare providers^1,2^.

A study describing the satisfaction of patients attending the general outpatient clinics of the National Hospital, Abuja found that patients who had long waiting times were not satisfied with the services^3^. The majority of doctors in Nigeria exhibit a strong commitment to their profession, diligently delivering high-quality healthcare services to their patients^4^. Doctors engage in intensive training and possess expertise in their respective speciality. Despite the high calibre training and clear commitment to providing standard healthcare services, certain factors impair effective service delivery. These include heavy patient case load, shortage of infrastructure, and consequently long working hours, which may occasionally have an influence on their professional demeanour^4^. It has been reported by the National Human Resources for Health Strategic Plan that the doctor to population ratio is 30:100 000 which imposes a huge burden on doctors^5^. The arduous working conditions and constrained resources can result in heightened levels of stress and burnout within the healthcare profession.

The doctor-patient relationship is a crucial aspect of healthcare delivery. Typically, medical practitioners in Nigeria uphold professional interactions with their patients. Nevertheless, there has been an increasing focus on the provision of patient-centred care, a paradigm that advocates for the active involvement of physicians in engaging patients in decision-making processes and enhancing communication channels^6^. Also, patients in Nigeria, akin to their counterparts in numerous other nations, hold the anticipation of receiving healthcare services that are prompt and efficacious. Patients desire their physicians to possess a high level of expertise, attentiveness, and compassion.

The trust that patients place in the healthcare system can vary significantly. There is a notable disparity in individual perspectives concerning the trustworthiness of healthcare providers and the healthcare system as a whole^7^. Some individuals harbour a high degree of confidence in their doctors and the overarching healthcare framework, often based on positive past experiences or a belief in the competence of their providers. Conversely, there is a segment of the population that harbours mistrust towards their doctors, a sentiment often rooted in previous unsatisfactory experiences or perceived inadequacies within the system^7^.

This scepticism might also originate from a lack of familiarity with doctors and other healthcare professionals, potentially leading to hesitance in establishing trust. Research indicates that fostering trust between patients and their healthcare providers is a process that often requires time and consistent positive interactions^8^. However, the existing medical record systems might not be sufficiently developed to promote continuous communication between a patient and a specific doctor over a prolonged period, which can be a significant barrier in building and maintaining trust.

A significant portion of the Nigerian population encounters financial constraints which is largely due to out-pocket spending and low level of insurance, which can pose obstacles in obtaining healthcare services^9^. Certain individuals may exhibit a tendency to postpone seeking medical care or choose alternative therapies based on cost considerations^9^. Cultural factors also play a significant role in shaping patients’ attitudes and behaviours towards healthcare delivery^9^.

The influence of traditional medicine and spiritual beliefs on patient decision-making and attitudes towards healthcare is noteworthy^9^. For example, the prevalent belief that mental illness is caused by spiritual possession or curses led to the use of alternative treatments such as traditional healing homes and faith-based institutions^10^. In the Nigerian context, a significant portion of the population encounters financial limitations, resulting in the potential imposition of a financial burden when seeking medical care^11^. Patients highly value physicians and medical professionals who offer affordable alternatives or engage in proactive discussions regarding potential expenses in order to prevent unforeseen financial burdens. The attention to affordability and cost-effective management plan is one that could likely increase patient compliance to treatment.

The provision of respect, dignity, and empathy by physicians is generally valued by patients. The importance of effective communication cannot be overstated, particularly when it comes to attentively listening to patients’ concerns and delivering concise and easily comprehensible explanations. Patients highly appreciate physicians who allocate sufficient time to comprehend their symptoms, elucidate the diagnosis and available treatment alternatives, and effectively address any inquiries or uncertainties they may possess^12^.

Patients anticipate that physicians will demonstrate a high level of expertise, competence, and knowledge with regards to contemporary medical advancements^13^. They desire to see their doctor’s adeptness in providing accurate diagnoses and appropriate treatments. A significant emphasis is placed on professionalism, which encapsulates a variety of attributes including punctuality, maintaining confidentiality, and adherence to ethical principles. Other critical factors influencing patients’ reactions to healthcare are availability and accessibility. Patients appreciate doctors who are accessible, which includes geographical proximity and the availability of appointments^14^. Extended waiting periods, limited accessibility to healthcare facilities, and difficulties in reaching physicians can foster frustration and dissatisfaction among patients.

The provision of high-quality care is highly valued by patients, as it guarantees optimal health outcomes for their medical conditions. This encompasses precise diagnoses, suitable treatment plan, and subsequent care. Patients have an inherent expectation that physicians remain abreast of current medical research, employ evidence-based methodologies, and demonstrate a steadfast dedication to ongoing education and enhancement^15^. The establishment of trust and confidence is crucial for patients, as it is imperative that they place their trust in their healthcare providers and possess a sense of assurance in their professional competencies. The establishment of a reliable doctor-patient relationship is predicated upon the principles of candid and transparent communication. Patients who have a high level of trust in their healthcare providers are more inclined to comply with treatment plans, adhere to prescribed medications, and report greater satisfaction with their overall healthcare encounters^16^.

The main limitation of this study is its observational nature. The attitude of health workers to the patients, which have limited studies, could affect the level of satisfaction of the patients. We suggest future research that will incorporate healthcare workers’ attitude into the factors determining patient satisfaction in Nigeria.

Patient satisfaction is an important outcome which reflects the quality of healthcare of which patient waiting time is an important component. It is imperative to acknowledge that these observations constitute generalizations and may not universally apply to all doctors or patients in Nigeria. The attitudes and responses exhibited by individuals, regions, and healthcare facilities within the country can exhibit variation. Furthermore, there are ongoing endeavours in Nigeria to enhance healthcare provision and fortify the doctor-patient rapport. These initiatives encompass training programs, infrastructure advancements, and community involvement. Understanding the nexus between patient perception and how it affects their response to healthcare therefore, will lead to delivery of a more patient-focused healthcare system and will ultimately improve healthcare outcomes

## Ethical approval

Ethical approval was not required for this Editorial.

## Consent

Informed consent was not required for this Editorial.

## Sources of funding

No funds, grants, or other support was received.

## Author contribution

A.T.A. conceptualized the editorial, and all the authors contributed to the writing, editing, and revising the final draft.

## Conflicts of interest disclosure

All authors declare that there is no conflict of interest.

## Research registration unique identifying number (UIN)

Not applicable.

## Guarantor

Abdullahi Tunde Aborode and Rawa Badri.

## Data availability statement

Not applicable.

## Provenance and peer review

The paper is not invited.
